# Drug-Free Enzyme-Based Bactericidal Nanomotors against Pathogenic Bacteria

**DOI:** 10.1021/acsami.1c00986

**Published:** 2021-03-26

**Authors:** Diana Vilela, Nuria Blanco-Cabra, Ander Eguskiza, Ana C. Hortelao, Eduard Torrents, Samuel Sanchez

**Affiliations:** † Smart nano-bio-devices, Institute for Bioengineering of Catalonia (IBEC), The Barcelona Institute of Science and Technology (BIST), Baldiri Reixac 10-12, 08028 Barcelona Spain; ‡ Bacterial infections: antimicrobial therapies, Institute for Bioengineering of Catalonia (IBEC), The Barcelona Institute of Science and Technology (BIST), Baldiri Reixac 10-12, 08028 Barcelona Spain; § Microbiology Section, Department of Genetics, Microbiology and Statistics Faculty of Biology, University of Barcelona, 643 Diagonal Ave., 08028 Barcelona, Spain; ∥ Institució Catalana de Recerca i Estudis Avancats (ICREA), Pg. Lluís Companys 23, 08010 Barcelona, Spain

**Keywords:** enzymatic nanomotors, biofilms, E. coli, infections, nanomachines, self-propulsion

## Abstract

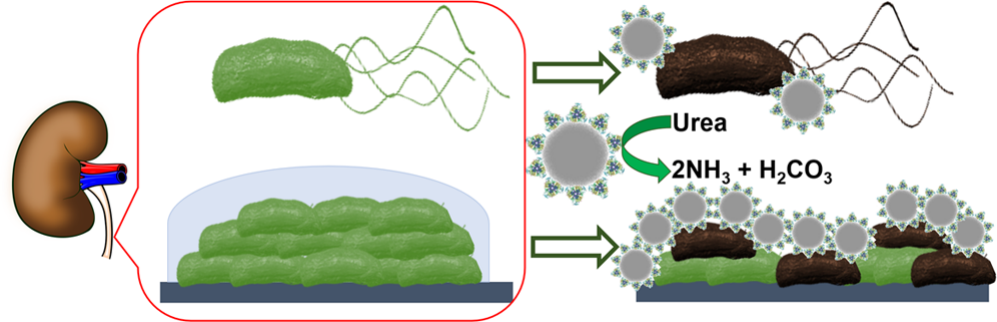

The low efficacy of current conventional treatments for bacterial infections increases mortality rates worldwide. To alleviate this global health problem, we propose drug-free enzyme-based nanomotors for the treatment of bacterial urinary-tract infections. We develop nanomotors consisting of mesoporous silica nanoparticles (MSNPs) that were functionalized with either urease (U-MSNPs), lysozyme (L-MSNPs), or urease and lysozyme (M-MSNPs), and use them against nonpathogenic planktonic *Escherichia coli*. U-MSNPs exhibited the highest bactericidal activity due to biocatalysis of urea into NaHCO_3_ and NH_3_, which also propels U-MSNPs. In addition, U-MSNPs in concentrations above 200 μg/mL were capable of successfully reducing 60% of the biofilm biomass of a uropathogenic *E. coli* strain. This study thus provides a proof-of-concept, demonstrating that enzyme-based nanomotors are capable of fighting infectious diseases. This approach could potentially be extended to other kinds of diseases by selecting appropriate biomolecules.

## INTRODUCTION

Bacterial infections are among the most common causes of morbidity and mortality in the world.^1^ In recent decades, the overuse of antibacterial agents has led to a growing risk of antibiotic-resistant bacterial infections, which have reached a level of prevalence that endangers public health and is becoming a major global concern as conventional therapies are losing efficacy.^2,3^ Conventional medicine urgently requires more sensitive technologies for imaging and early detection, new methods for accurate and early diagnosis, better pharmaceutical properties of drugs (stability, solubility, circulation time, and localized accumulation), and the capacity to target and control drug release to minimize adverse side-effects.^4^ Any advances in this field hold a great promise for improving the quality of life and survival of patients and will lead the way to more personalized medicine.

Nanomedicine is experiencing rapid growth due to its potential for monitoring and treating physiological conditions using nanoscale devices such as particles, materials, and drug delivery systems (DDS).^5,6^ Nanomaterials possess structural properties that enable them to serve as potential noninvasive tools for diagnostic imaging, disease detection, and efficient drug delivery, thereby improving drug solubility and specificity, which provides new opportunities to improve the safety and efficacy of conventional therapeutics.^7^ However, one of the greatest challenges that determine the success of nanomaterials (incl. nanoparticles) is their ability to reach the therapeutic site and deliver the necessary doses while minimizing accumulation at undesired sites due to the body’s biological barriers (immune clearance, permeation across the endothelium, penetration through tissues and endocytosis into the target cells).^8,9^

Micro/nanomotors and micro/nanoscale devices are designed to perform specific mechanical movements in response to certain stimuli. They are promising platforms that offer rapid drug transportation, high tissue penetration, and control of motion.^10−12^ Recent studies successfully demonstrated that compared to passive DDS, micro/nanomotors provide improved drug diffusion and delivery to target locations.^11,13−18^ Enzyme-powered micromotors^19,20^ are chemically powered and have great potential as they can “run” on physiologically available fuels such as glucose,^21,22^ triglycerides,^23,24^ and urea.^17,25−27^ Due to their versatility, micro/nanomotors are being used more ubiquitously for treating a growing number of diseases including diabetes,^28^ cancer,^29−31^ and bacterial infections.^17,32−38^ For instance, Esteban-Fernández et al. developed chitosan-based bactericidal micromotors using water-soluble metals (magnesium), where the production of hydrogen gas in gastric acid media delivers the necessary propulsion.^39^ The same group also provided the first evidence of a successful *in vivo* drug delivery using micromotors, more specifically, to treat a gastric bacterial infection in a mouse model.^32^ Stanton et al. demonstrated that nonpathogenic magnetotactic bacteria (MSR-1) can be integrated into drug-loaded mesoporous silica microtubes to obtain controllable microswimmers (biohybrids) capable of targeted delivery of antibiotics to an infectious biofilm.^33^ Tang et al. transformed passive cells into active cell robots through a design involving enzyme-powered Janus platelet cell robots for active and targeted delivery of antibiotics against the Gram-negative *Escherichia coli*.^17^ More recently, magnetotactic T-Budbots were designed deploying antibiotic-laden magnetic tea buds against biofilms of *Pseudomonas aeruginosa* and *Staphylococcus aureus*.^35^ Furthermore, tubular catalytic microrobots have demonstrated a high antibacterial activity when used to degrade dental biofilm in the presence of 1% H_2_O_2_.^36^ However, despite the fast growth in the nanomotors field over the past few years, their application as bactericidal tools has been rarely explored, and if so, nanomotors release antibiotics to kill the bacteria, not making use of the chemical reaction that propels them also for that aim.

In this study, we develop the first drug-free enzyme-based mesoporous silica nanomotors capable of killing bacteria while swimming on a biological fuel, which should minimize drug-related side-effects. Mesoporous silica nanoparticles (MSNPs) were synthesized and their surface was modified using glutaraldehyde with either urease (U-MSNPs), lysozyme (L-MSNPs), or a combination of urease and lysozyme (M-MSNPs). We then evaluated the bactericidal efficacy of each type of functionalized nanomotor (in the presence of urea) against two types of bacteria: (i) nonpathogenic planktonic bacteria *E. coli*, and (ii) a biofilm of a uropathogenic *E. coli*, which is typically involved in urinary-tract infections. We also tested the bactericidal capacity of bicarbonate and ammonia, both enzymatic products of urease, to evaluate the antibacterial nature of urease. Finally, we studied the movement of urease-based nanomotors in phosphate-buffered saline (PBS), Lysogeny broth (LB), and simulated urine.

## Results and Discussion

### Characterization of Enzyme-Based MSNPs

**Figure 1:**
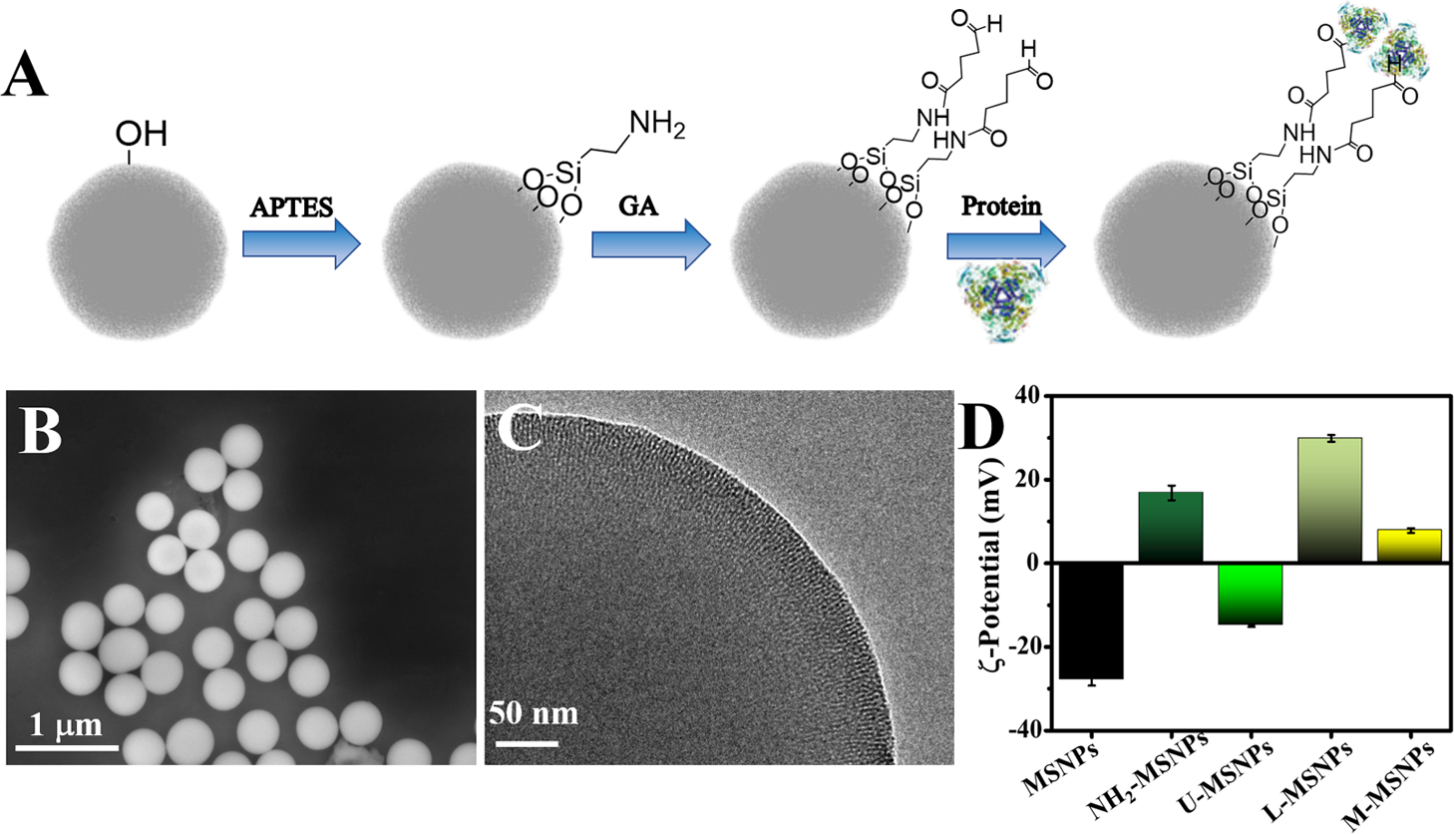
Fabrication and characterization of enzyme-based mesoporous silica nanoparticles. (A) Scheme of the stepwise fabrication process to synthesize enzyme-based nanomotors. (B) Scanning electron microscopy (SEM) image of mesoporous silica nanoparticles (MSNPs). (C) TEM image of MSNPs showing the porous particle surface. (D) Surface charge of the unmodified MSNPs, the amino-modified MSNPs (NH_2_-MSNPs), the urease-modified MSNPs (U-MSNPs), the lysozyme-modified MSNPs (L-MSNPs), and the urease- and lysozyme-modified MSNPs (MMSNPs) (*N* = 3, error bars indicate SE).

Mesoporous silica nanoparticles (MSNPs) were synthesized via sol–gel chemistry.^40^ In order to obtain the desired porosity, a surfactant (cetyltrimethylammonium bromide [CTAB]) was used as a pore template and triethanolamine (TEOA) was used as a base catalyst. The as-prepared MSNPs were functionalized with (3-aminopropyl)triethoxysilane (APTES) and subsequently with proteins, either urease, lysozyme, or a combination of both, to fabricate the enzyme-based nanomotors (Figure 1A).

The as-prepared MSNPs were characterized by scanning electron microscopy (SEM) (Figure 1B) and transmission electron microscopy (TEM) (Figure 1C). SEM analysis was used to determine the diameter of the as-prepared MSNPs to be 411 ± 11 nm (average ± one standard deviation, *n* = 50), and confirm a high level of monodispersity (polydispersity index of 0.02). Moreover, the TEM image showed the porous structure of MSNPs, revealing a radial pattern (Figure 1C). In a previous study, we estimated the pore diameter of these MSNPs as 2 nm using Brunauer–Emmett–Teller (BET) analysis.^40^

For the functionalization of the as-prepared MSNPs with different proteins, their hydroxyl moieties were first modified with amino groups before activating them with aldehyde groups using aminopropyltriethoxysilane (APTES) and glutaraldehyde (GA), successively. Finally, glutaraldehyde, as a linker, was used to facilitate the modification of the MSNP surface along with the reaction of the aldehyde terminal groups of the MSNPs and the amino moieties from the proteins. Each step of the MSNP functionalization was monitored using dynamic light scattering (DLS) (Figure 1D), while the amount of protein linked to the particle was monitored using a commercial kit based on Coomassie brilliant blue G (Figure S1A). The electrophoretic mobility analysis of MSNPs indicated a negative surface charge of −28.0 ± 1.3 mV (average ± 1 SD, *N* = 5, Figure 1D), typical for the −OH moieties on the as-prepared MSNPs. Once the MSNPs were modified with APTES, the surface charge changed and became positive: 16.8 ± 1.8 mV, which indicates the presence of amine groups and, as a consequence, confirms the success of the modification process.

The last functionalization step for the synthesis of the protein-based MSNPs is the covalent attachment of either urease (U-MSNPs), lysozyme (L-MSNPs), or a combination of both (M-MSNPs) using measured changes in the electrical charge of MSNPs to verify the successful attachment of each type of protein (Figure 1D). Given the isoelectric points (pI) of each enzyme, pI (urease) = 4.9^41^ and pI (lysozyme) = 10.7,^42^ the surface charges measured at pH 7.4 using DLS, namely −14.9 ± 0.3 mV (average ± 1 SD, *N* = 5) for U-MSNPs, 29.9 ± 0.8 mV (*N* = 5) for L-MSNPs, and 7.8 ± 0.6 mV (*N* = 5) for M-MSNPs were in agreement with the surface charge of the free proteins at pH 7.4. In addition, to demonstrate that the different proteins successfully bound to the MSNP surfaces, we quantified them using a colorimetric method for proteins (Figure S1A, see the Experimental Methods section for details). The amounts of protein bound to the MSNPs (1 mg/mL) were obtained using linear interpolation: 153.2 ± 15.4, 71.5 ± 0.2, and 94.8 ± 5.4 μg/mL (average ± 1 SE, *N* = 6) for U-MSNPs, L-MSNPs, and M-MSNPs, respectively. Furthermore, we tested for the presence of bound urease in U-MSNPs and M-MSNPs using a kit that quantifies the activity of the urease enzyme (Figure S1B). As expected, L-MSNPs did not show any urease activity, while U-MSNPs showed higher activity compared to M-MSNPs since the amount of urease on the M-MSNP surface is lower than that for U-MSNPs. Since protein-based MSNPs are often used after having been in storage for several days, we also studied the effect of storage (at 4 °C for up to 14 days) on urease activity (Figure S2). During the first week of storage, the loss of urease activity in both U-MSNPs and M-MSNPs was below 20%. During the second week, this loss remained below 40%, which means that they are still capable of fulfilling their purpose even 14 days after fabrication.

### Bactericidal Capacity of U-MSNPs, L-MSNPs, and M-MSNPs

The bactericidal enzymes urease and lysozyme were selected for the modification of MSNPs to obtain protein-based nanomotors that could be used against pathogenic bacteria. Lysozyme is a well-known antimicrobial enzyme that kills bacteria by the hydrolysis of the 1,4-β-linkages between *N*-acetylmuramic acid and *N*-acetyl-d-glucosamine residues in peptidoglycan from the cell wall.^34,43^ Urease is an enzyme that can catalyze the hydrolysis of urea and induce the death of *E. coli* (of both the nonpathogenic and pathogenic strains) as a result of producing carbonate and ammonia generating an alkaline pH.^44−47^ To demonstrate that NH_4_^+^ and HCO_3_^–^, both enzymatic products of urea hydrolysis by urease, can kill *E. coli,* we incubated *E. coli* (1 × 10^8^ cells/mL) with NH_4_^+^ and HCO_3_^–^ at concentrations of 10, 30, and 50 mM for 1 h. Then, cells were treated with propidium iodide and STYO 9 and imaged using a fluorescence microscope (Figure S3A). By identifying and counting the number of dead and live bacteria, we could estimate the bactericidal efficacy of each incubation (Figure S3B). While both NH_4_^+^ and HCO_3_^–^ exhibited a bactericidal capacity that increased with increasing concentration, the overall efficacy was higher with NH_4_^+^. Urease should therefore be the preferred choice for fabricating bactericidal enzyme-based nanomotors.

**Figure 2:**
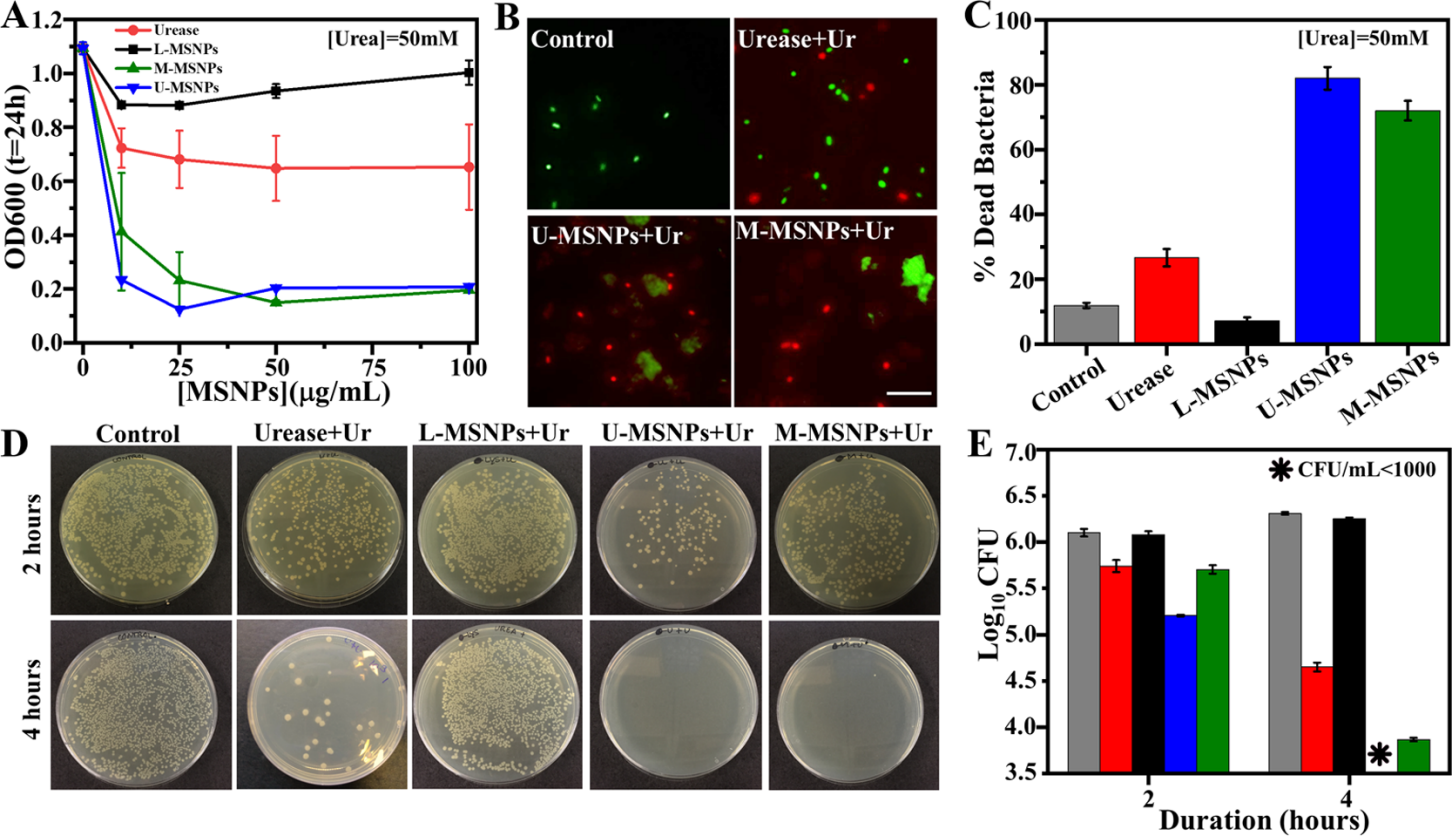
Evaluating the bactericidal capacity of the different enzyme-based micromotors: (A) optical density (OD_600_) of nonpathogenic *E. coli* after 24 h in the presence of different concentrations of urease, U-MSNPs, L-MSNPs, and M-MSNPs. (B) Fluorescence images and (C) percentage of dead bacteria determined by live/dead assay after 2 h of 1 × 10^8^ CFU/mL *E. coli* treated with 12.5 μg/mL (minimum inhibitory concentration, MIC_50_) for urease, U-MSNPs, L-MSNPs, and M-MSNPs. (D) *E. coli* counts (log 10 CFU/mL) after 2 and 4 h of treatment with 12.5 μg/mL (MIC_50_) urease, U-MSNPs, L-MSNPs, and M-MSNPs. (E) Photographs of Petri plates at 10^3^ CFU dilution used to measure the efficacy of urease, U-MSNPs, L-MSNPs, and M-MSNPs against *E. coli* after 2 and 4 h. All experiments were carried out at [urea] = 50 mM (*N* = 3, error bars represent SE).

The bactericidal capability of enzyme-based MSNPs was evaluated by incubating nonpathogenic *E. coli* with each type of MSNP (Figure 2) at optimal urea concentrations.^29^ First, we estimated the minimum inhibitory concentration (MIC_50_) of each enzyme-based MSNP for killing nonpathogenic *E. coli* by incubating different concentrations (0–100 μg/mL) of each MSNP for 24 h with a certain concentration of cells. The optical density (OD_600_) (Figure 2A) of *E. coli* after 24 h indicated that 12.5 μg/mL was the MIC_50_ for U-MSNPs and M-MSNPs but not for L-MSNPs, which were unable to kill *E. coli* at the chosen concentration range. Then, taking 12.5 μg/mL as a reference concentration of enzyme-based MSNPs, we incubated *E. coli* with the selected U-MSNP, L-MSNP, and M-MSNP concentrations (including controls without any MSNPs) and monitored the number of live and dead cells using fluorescence live/dead assay (Figures S5 and S6). While samples without urease activity (i.e., no urease or urea present) did not exhibit any bactericidal capability, all samples that contained urease activity displayed a bactericidal ability that was highest with U-MSNPs (Figure 2B,C). These results are supported by *E. coli* counts (log 10 CFU/mL) after 2 and 4 h of treatment with 12.5 μg/mL of each MSNP (Figures S7–S9). As before, only samples containing urease activity exhibited any bactericidal capabilities (Figure 2D,E) with U-MSNPs showing the highest efficacy with 82% dead bacteria (from fluorescence assay, Figure 2C). We, therefore, selected U-MSNPs for the experiments that test the ability of MSNPs to fight urinary-tract bacterial infections. It is worth pointing out that neither lysozyme nor L-MSNPs showed any bactericidal behavior. This is in agreement with earlier reports that suggested that lysozyme by itself can lyse Gram-positive bacteria, but for Gram-negative bacteria, as *E. coli*, it needs help from other factors such as ethylenediamine tetraacetic acid (EDTA) or complement that enable lysozyme to penetrate the outer membrane (Figure S4).^48,49^

**Figure 3:**
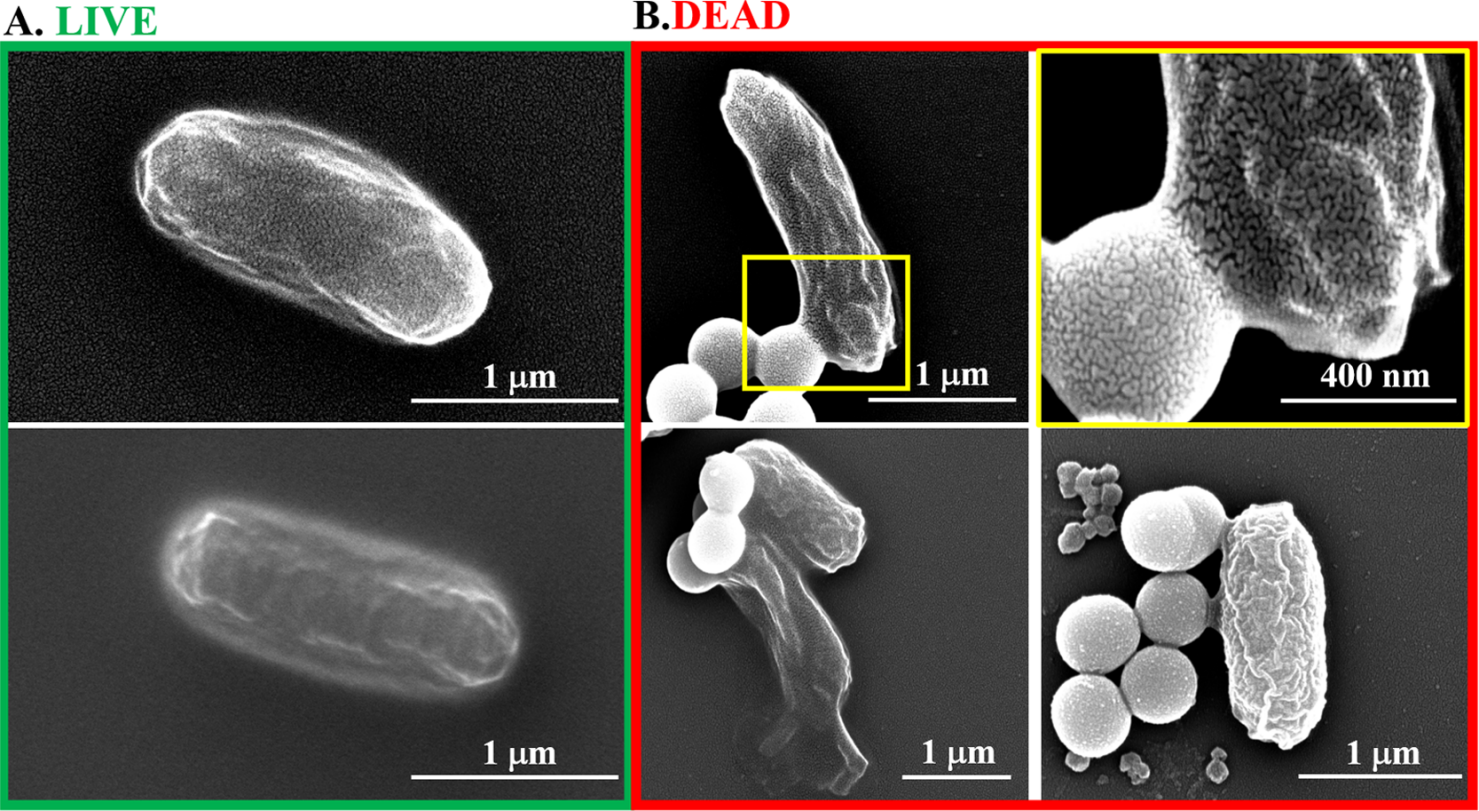
Bacteria imaged with SEM: Examples of (A) live *E. coli* MG1655; (B) dead bacteria after having been treated with U-MSNPs for 2 h in the presence of 50 mM urea. Yellow box depicts a zoom image of (B) the bacteria in the top row.

Using SEM, we then imaged the bacteria before and 2 h after treatment with U-MSNP nanomotors in the presence of 50 mM urea (Figure 3). Figure 3B illustrates how the U-MSNP nanomotors attached to the *E. coli* surface while trying to penetrate the cell, and how the nanomotors destroyed some cell bodies because of the production of bicarbonate and ammonia. These results suggest how U-MSNP nanomotors kill *E. coli*, possibly due to synergistic effects between diffusion (which increases contact with bacteria) and the enzymatic reaction that occurs on the nanomotor surface in the presence of the particular substrate.

**Figure 4:**
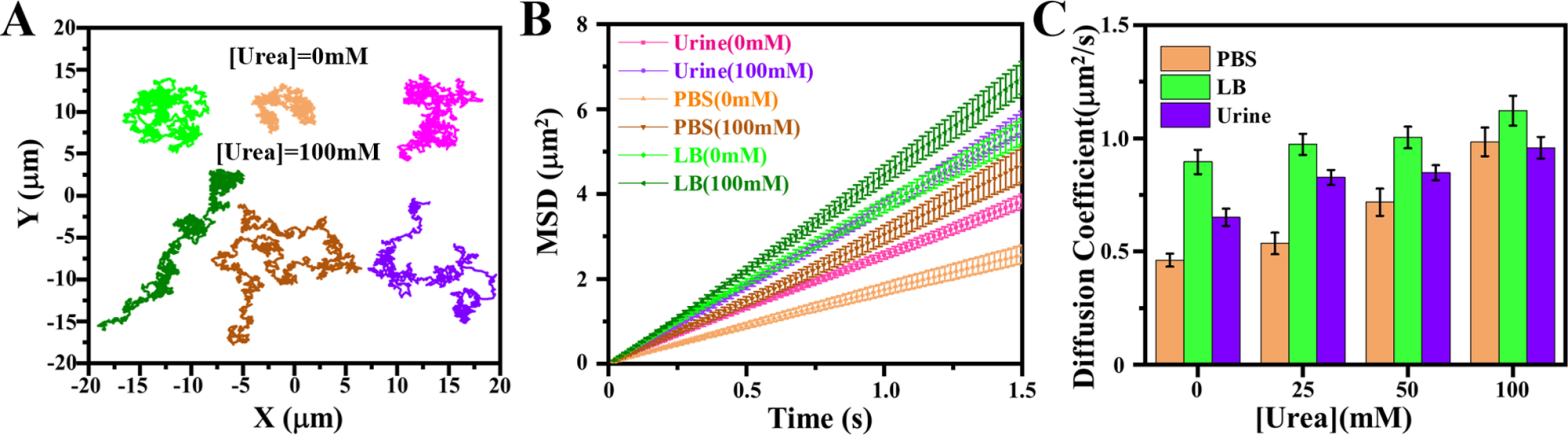
Motion analysis of urease-based nanomotors (U-MSNPs) in PBS, LB, and simulated urine. (A) Representative trajectories of U-MSNPs at 0 mM (top) and 100 mM urea (bottom). (B) Mean-squared displacements (MSDs) of U-MSNPs at 0 and 100 mM. (C) Effective diffusion coefficients calculated from the MSDs at different urea concentrations (*N* = 20, error bars show SE).

We also assessed the motility of U-MSNP nanomotors in different media: PBS, LB, and simulated urine (Figure 4). Previous studies have shown that the presence of a simple geometrical asymmetry can propel micro- and nanostructures at low Reynolds numbers as these asymmetries cause an asymmetrical generation of forces.^50,51^ Based on these findings, we showed in an earlier publication how directional self-propulsion can be achieved using non-Janus spherical micromotors powered by enzyme catalysis simply by controlling enzyme distribution and quantity.^51^ Taking into account that U-MSNP nanomotors possess an intrinsic asymmetry due to the way that enzymes bind to their surface,^52^ we studied the motion of these nanomotors at different urea (enzyme–substrate) concentrations (0, 25, 50, and 100 mM). We tracked the trajectories of some U-MSNP nanomotors over a 30 s period, both in the absence and presence of urea (100 mM) (Figure 4A and Videos S1–S3), and used these trajectories to calculate the mean-squared displacement (MSD) (Figure 4B). The MSD has a steeper slope in the presence of urea and shows a linear trend over time. We obtained the effective diffusion coefficient, De, from fitting the MSDs of each trajectory to
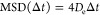
1We also observed both a media type- and substrate concentration dependence of diffusion with diffusion generally increasing with higher substrate concentrations (Figure 4C).

**Figure 5:**
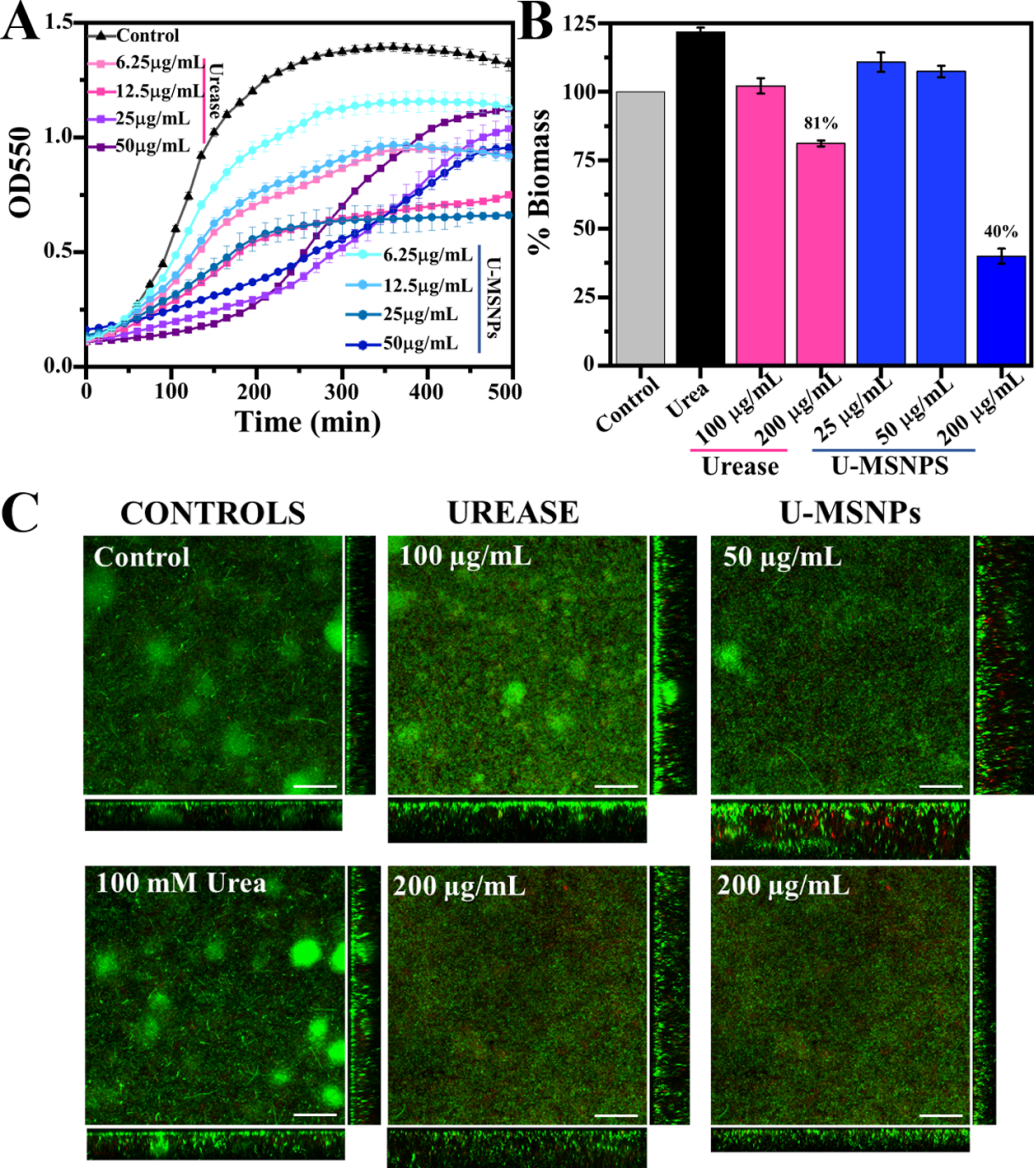
Evaluating the bactericidal capacity of urease-based nanomotors (U-MSNPs) against uropathogenic *E. coli* (CFT073): (A) optical density (OD_550_) of planktonic uropathogenic *E. coli* for different concentrations of urease and U-MSNPs; (B) percentage of the biofilm biomass from uropathogenic *E. coli* remaining after treatment with U-MSNP nanomotors and excess of urease (at 5- to 10-fold, the highest U-MSNP nanomotor concentrations applied); and (C) simulated fluorescence projections and orthogonal view sections of 4-day uropathogenic *E. coli* biofilm before and 6 h after treatment with different concentrations of urease and U-MSNPs (scale bar = 50 μm). All experiments were carried out at [urea] = 100 mM. (*N* = 3, error bars represent SE).

Finally, to demonstrate that U-MSNP nanomotors can kill pathogenic *E. coli* and be efficient tools for treating urinary-tract infections, we studied their antibacterial capacity on a uropathogenic *E. coli* strain (CFT073) in planktonic and biofilm states (Figure 5).^53^ First, we estimated the MIC_50_ of U-MSNPs nanomotors vs excess of urease (free-enzyme) for killing planktonic uropathogenic *E. coli*. The OD_550_ analysis yielded an MIC_50_ of U-MSNPs nanomotors against uropathogenic *E. coli* of 25 μg/mL (Figure 5A). Based on this result, we tested the efficacy of different U-MSNP nanomotor concentrations (25, 50, and 200 μg/mL) to disrupt uropathogenic *E. coli* biofilms (Figure 5B,C). We found that uropathogenic *E. coli* biofilms were not disrupted by U-MSNP nanomotor concentrations below 200 μg/mL (the same threshold was found for the free-enzyme). While U-MSNPs at 200 μg/mL reduced the biofilm’s biomass by 60%, the excess of the free-enzyme (10-fold) only achieved a biomass reduction of 19%. Thus, U-MSNP nanomotors at a concentration of 200 μg/mL should be much more efficient at battling urinary-tract infections than the free enzyme.

## Conclusions

In this study, we demonstrate that urease-based nanomotors are efficient tools against urinary-tract infections due to the localized production of urease enzymatic products on the surface of U-MSNP nanomotors and their high diffusivity, which increases contact with the bacteria. First, we synthesized and characterized three types of enzyme-based MSNPs: U-MSNPs, L-MSNPs, and M-MSNPs. We then tested their bactericidal capacity on planktonic *E. coli*. Such a capacity was found for U-MSNPs and M-MSNPs due to the presence of urease enzymatic products, with U-MSNPs proving more effective. Finally, we tested the effect of different concentrations of U-MSNPs on their bactericidal efficacy against a planktonic pathogenic *E. coli* strain, which is often involved in urinary-tract infections. We found that they start to become highly effective at relatively low concentrations of 200 μg/mL. Such enzyme-based nanomotors thus represent a viable alternative for treating infectious diseases.

## Experimental Methods

### Materials

Ethanol (EtOH, 99%), methanol (MeOH, 99%), hydrochloric acid (37% in water), ammonium hydroxide (NH_4_OH, 25% in water), tetraethylorthosilicate (TEOS, 99%), triethanolamine (TEOA, 99%), cetyltrimethylammonium bromide (CTAB, 99%), ammonium nitrate (NH_4_NO_3_), bicarbonate (NaHCO_3_), 3-aminopropyltriethoxysilane (APTES, 99%), glutaraldehyde (GA, 25% in water), urease (from Canavalia ensiformis, Type IX, powder, 50 000–100 000 units/g solid), lysozyme (100 kU/mg, Orion High Technologies), Urease Activity Assay Kit (MAK120, Sigma-Aldrich), Protein Quantification Kit (51254, Sigma-Aldrich), urea (99.9%), potassium dihydrogen phosphate (KH_2_PO_4_), dibasic potassium phosphate (K_2_HPO_4_), Phosphate buffer saline (PBS, pH 7.4). *Micrococcus lysodeikticus* (ATCC No. 4698, M3770 Sigma-Aldrich), uropathogenic *E. coli* (UPEC) CFT073 strain (ATCC 700928) and nonpathogenic *E. coli* strain MG1655 (ATCC 700926), LB broth, LB broth with agar, hexamethyldisilazane (HMDS, Sigma-Aldrich), LIVE/DEAD BacLight Bacterial Viability Kit (L7007, ThermoFisher) have been employed.

### Equipment

Scanning electron microscopy (SEM) images were captured using a FEI NOVA NanoSEM 230 at 5 kV. Transmission electron microscopy (TEM) images were captured using a JEOL JEM-2100 microscope. The ζ-potential and hydrodynamic radius were measured using a Malvern Zetasizer Nano ZS system. Protein quantification, enzymatic activity assays, and OD_600_ determination were carried out using a Synergy HTX Absorbance microplate reader and a Synergy H1M Fluorescence microplate reader. A spectrophotometer Specord 50/plus (Analytik Jena, Germany) was employed to monitor the U-MSNP and M-MSNP activity for 14 days. Optical videos were recorded using an inverted optical microscope (Leica DMi8) equipped with a 63× water objective. Fluorescence images of live/dead assay were acquired using an inverted optical microscope (Leica DMI3000B), coupled with a 10×, 20×, 40×, and 63× objectives, along with a Leica digital camera DFC3000G with LAS V4.5 software. The videos were analyzed using Python-based code. Growth curves of planktonic *E. coli* were performed using a SPARK Multimode microplate reader (Tecan). Continuous biofilms were imaged using a Zeiss LSM 800 confocal laser scanning microscope (CLSM) with a 20×/0.8 air objective. FIJI and COMSTAT2 software were used for biofilm biomass quantification. Origin 2018, Microsoft Excel Professional, and ImageJ were employed for the analysis of the experimental data.

## Experimental Procedure

### Synthesis of Urease (U-MSNPs), Lysozyme (L-MSNPs), and Urease and Lysozyme (M-MSNPs)

#### Synthesis of Mesoporous Silica Nanoparticles (MSNPs)

MSNPs were prepared using a sol–gel method. Briefly, a solution containing CTAB (570 mg), TEOA (35 g), and water (20 mL) was heated to 95 °C in a silicon oil bath. This mixture was stirred for 30 min, and subsequently, TEOS (1.5 mL) was added dropwise. The mixture was further stirred at 95 °C for 2 h. The produced particles were collected by centrifugation and washed with ethanol (3 times, 3500 rpm, 10 min). For removal of CTAB from the MSNP pores, the particles were suspended in EtOH (60 mL) and ammonium nitrate (160 mg) and heated at 60 °C for 1 h. Finally, the particles are collected by centrifugation, washed in ethanol (3 times, 3500 rpm, 10 min), and sonicated for 10 min between each centrifugation. To determine the concentration of the MSNP suspension, 3 aliquots (0.5 mL) were collected, centrifuged, and air-dried at 70 °C.

#### Amine Functionalization of MSNPs (MSNP-NH_2_)

The previously synthesized MSNPs were suspended in MeOH (1 mg/mL). Then, APTES was added to the suspension (1% V/V) and it was shaken for 24 h at room temperature, using a rotating wheel Eppendorf shaker. Finally, the particles were collected by centrifugation, washed first in ethanol 3 times (3500 rpm, 5 min) and then in water 3 times (3500 rpm, 10 min), and sonicated for 10 min between each centrifugation. To determine the concentration of the MSNPs-NH_2_ suspension, 3 aliquots (0.5 mL) were collected, centrifuged, and air-dried at 70 °C.

#### Functionalization of MSNP-NH_2_ with Urease (U-MSNPs), Lysozyme (L-MSNPs), and Urease and Lysozyme (M-MSNPs)

MSNP-NH_2_ (1 mg/mL) were centrifuged at 3500 rpm for 5 min, washed twice with PBS, suspended in 900 μL of PBS, and sonicated for 10 min. After that, 100 μL of glutaraldehyde (GA) was added, and the mixture was well-dispersed. The mixture was placed on a rotating wheel Eppendorf shaker for 3 h at room temperature. GA-MSNPs were then collected and washed three times with PBS (3500 rpm, 5 min) and sonicated for 10 min between each wash. Next, the GA-MSNPs were suspended in PBS containing 3 mg/mL urease, lysozyme or urease, and lysozyme, respectively. Then, the mixture was placed on a rotating wheel Eppendorf shaker overnight at 4 °C. The resulting modified nanomotors were washed three times with PBS by centrifugation (3500 rpm, 5 min), intercalating the washes with 1 min of sonication.

### Bacteria Culture and Biofilm Growth

#### Bacteria Culture

*E. coli* MG1655 cultured on LB agar plates were transferred to 5 mL LB broth and allowed to divide overnight at 37 °C and 200 rpm. The overnight MG1655 culture (0.5 mL) was diluted in 5 mL of fresh LB broth and allowed to grow another 2 h. To estimate the bacterial concentration, the optical density was measured at 600 nm (OD_600_). For the evaluation of the activity of protein modified-MSNPs against *E. coli*, bacteria were centrifuged (6500 rpm, 3 min) and resuspended twice in PBS (pH 7.4). Bacteria were diluted to a determined concentration depending on the assay used.

*E. coli* on U-MSNPs were imaged using scanning electron microscopy (SEM, NOVA NanoSEM 230) at 5 keV. To prepare samples for SEM, each aliquot was suspended in motility media and allowed to sediment on clean plasma-etched (1 min argon plasma, Diener Electronic Atto Plasma Cleaner, Ebhausen, Germany) silicon wafer chips (5 × 6 mm) for 1 h at room temperature. Wafers were incubated in 2.5% glutaraldehyde in PBS for 45 min at 4 °C, rinsed with PBS, and then with water. Bacteria were dehydrated in a series of increasing aqueous ethanol concentrations (30, 50, 70, 90, and 100%) for 5 min in each solution and 10 min in pure ethanol. Bacteria were further dehydrated and preserved using a series of hexamethyldisilazane (HMDS, Sigma-Aldrich) solutions: 2:1 ethanol/HMDS (15 min), 1:2 ethanol/HMDS (15 min), and pure HMDS (15 min). Wafers were air-dried followed by sputtering deposition of 5 nm gold using a sputter Leica EM ACE600 coating system.

#### Biofilm of Uropathogenic E. coli Strain CFT073 Growth

Continuous biofilm of uropathogenic *E. coli* CFT073 growth was performed using a Flow-Cell system, as previously described,^54^ with some modifications. Briefly, after sterilizing the Flow-Cell system, 350 μL of an early exponential-phase culture of *E. coli* CFT073 (OD_600_ = 0.1) were inoculated into the Flow-Cells (DTU Systems Biology) and allowed to attach to the glass surface for 2 h. Afterward, media (0.1 × LB broth supplemented with 0.002% glucose) was supplied to the system at 42 μL/min using an Ismatec ISM 943 peristaltic pump (Ismatec). Bacteria were allowed to grow in biofilms for 96 h so that a mature biofilm could be established.

### Video Recording

#### Optical Video Recording of Nanomotors (U-MSNPs) and MSD Analysis

An inverted microscope equipped with a 63× water objective and a Hamamatsu camera was used to observe and record videos of the nanomotors’ movement. Samples of aqueous solutions of PBS, LB, and simulated urine containing U-MSNPs were placed, respectively, on a glass slide and mixed well with different concentrations of urea (0, 25, 50, 100 mM). The samples were then covered with a glass slide to avoid artifacts caused by drifting, and videos of 30 s at 50 frames per second using bright field were recorded. At least 20 U-MSNPs were tracked per condition. The videos were analyzed using Python-based code to obtain the trajectories of the nanomotors and calculate the mean-squared displacement (MSD) using the following equation
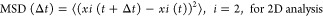
2After this, the diffusion coefficient (*D*_e_) was obtained by fitting the MSD data to eq 1, which is valid at short time intervals for small particles, with low rotational diffusion.^55^

### Protein Quantification and Activity Assays

#### Protein Quantification Assay

The quantification of the total protein attached to the U-MSNPs, L-MSNPs, and M-MSNPs was determined using a commercial kit based on Coomassie brilliant blue G, which interacts with proteins and stains blue under acidic conditions. The initial concentration of each sample was 1 mg/mL, and the experiment was performed according to the manufacturer’s instructions. The results were acquired by measuring the absorbance at 570–600 nm.

#### Urease Activity Assay

Enzymatic activity of U-MSNPs and M-MSNPs was evaluated using a commercial kit that determines the concentration of ammonia generated by Berthelot’s method. The nanomotors were at a concentration of 1 mg/mL, and the experiment was performed according to the manufacturer’s instructions. The results were acquired by measuring the absorbance at 670 nm.

#### Activity of U-MSNPs and M-MSNPs for 14 Days

The activity was calculated by the quantification of ammonia production by U-MSNPs and M-MSNPs, respectively, using a titration method. For this, 50 μg/mL of each type of nanomotor was incubated with 100 mM urea in a total volume of 1 mL. Then, 50 μL of *p*-nitrophenol was added to each sample and allowed to mix using a rotating wheel Eppendorf shaker for 30 min. Afterward, the samples were centrifuged, and the supernatants were transferred, respectively, to 5 mL vials for their titration with 10 mM HCl. The volumes required for the neutralization of each sample were acquired from the notebook.

### Evaluation of Bactericidal Activities

#### Evaluation of the Bactericidal Capability of NH_4_^+^ and HCO_3_^–^

Aliquots of nonpathogenic *E. coli* strain MG1655 (1 × 10^8^ cells/mL) were incubated with different concentrations (10, 30, and 50 mM) of urease enzymatic products (NH_4_^+^ and HCO_3_^–^) for 1 h. Then, the samples were washed 3 times with PBS (pH 7.4) and incubated with 1 μL/mL propidium iodide and STYO 9 (Life Technologies) for 10 min with gentle shaking. Then, they were washed twice with PBS (pH 7.4) and immediately imaged with a fluorescent microscope. Cell viability percentage was defined as the total number of live cells divided by the sum of live and dead cells using Image J software.

#### Evaluation of the Bactericidal Capability of Lysozyme and L-MSNPs at Different pH Values (5, 6, 7, 8, 9)

On the one hand, different concentrations of lysozyme (100, 10, 5, 2.5, and 1.25 μg/mL) were incubated with *M. lysodeikticus* (0.1 mg/mL). On the other hand, lysozyme and L-MSNPs (50, 25, and 12.5 μg/mL) were incubated with the nonpathogenic *E. coli* (1 × 10^8^ cells/mL), respectively. For both cells, incubation was carried out for 2 h at 37 °C and 200 rpm with different phosphate buffers (pH 5–9) by triplicate. Afterward, the samples were washed 3 times with PBS (pH 7.4) and incubated with 1 μL/mL propidium iodide and STYO 9 (Life Technologies) for 10 min with gentle shaking. Then, they were washed twice with PBS (pH 7.4) and immediately imaged with a fluorescent microscope. Percent cell viability was defined as the total number of live cells divided by the sum of live and dead cells using Image J software.

#### Calculation of MIC_50_ (Minimum Inhibitory Concentration)

About 1 × 10^6^ cells/mL of nonpathogenic *E. coli* were incubated (37 °C, 200 rpm) for 24 h at different concentrations of U-MSNPs, L-MSNPs, and M-MSNPs (0, 10, 25, 50, 100, 200, 300, and 500 μg/mL) in the presence of 50 mM urea and in the LB medium using 96-well plate (*n* = 3). As a control, in parallel, the same quantities of free urease in the presence of 50 mM urea and free lysozyme (without urea) were tested. Each well has a total volume of 200 μL. OD_600_ measurements were taken every 2 min for 24 h to establish the speed of proliferation and shape of the bacterial growth curve.

#### Evaluation of Bactericidal Capability of Protein-Modified MSNPs

About 1 × 10^8^ cells/mL of nonpathogenic *E. coli* MG1655 were incubated (37 °C, 200 rpm, PBS 7.4) for 2 and 4 h with 12.5 μg/mL U-MSNPs, L-MSNPs, and M-MSNPs, respectively, in the absence and presence of 50 mM urea in a total volume of 5 mL (*n* = 3). The same protocol was carried out for the free enzymes. After 2 and 4 h, an aliquot (1 mL) of each sample was taken and washed twice with PBS 7.4.

##### Live/Dead Assay

The samples were incubated with 1 μL/mL propidium iodide and STYO 9 (Life Technologies) for 10 min with gentle shaking. Then, they were washed twice with PBS (pH 7.4) and immediately imaged with a fluorescent microscope. Cell viability percentage was defined as the total number of live cells divided by the sum of live and dead cells using Image J software.

##### CFU Assay

The aliquots were serially diluted two times to obtain a final 1 × 10^5^ and 1 × 10^4^ CFU/mL concentration. Then, 100 μL of each dilution were cultured in LB agar plates and allowed to grow overnight at 37 °C. Bacterial concentration represents 10-fold of all colonies counted per plate since 0.1 mL were cultured.

#### Evaluation of the Bactericidal Capability of U-MSNP Nanomotors against Planktonic Pathogenic *E. coli* CFT073

About 200 μL of an early exponential-phase culture of *E. coli* CFT073 (OD_600_ = 0.1) was plated in a microtiter plate (Corning 3596 Polystyrene Flat Bottom 96 Well) mixed with different concentrations of U-MSNPs and urease (6.25, 12.5, 25, and 50 μg/mL). Then, 100 mM of urea was added, and the microtiter plate was incubated in the microplate reader at 37 °C and 150 rpm shaking. The growth of the bacteria was then monitored for 8 h by taking the absorbance (OD_550_) every 15 min. Minimal inhibitory concentration (MIC_50_) was defined as the concentration that reduces bacterial growth (OD_550_) by 50%.

#### Evaluation of the Bactericidal Capability of U-MSNP Nanomotors against Biofilm Pathogenic *E. coli* CFT073

Mature biofilms of *E. coli* CFT073 grown in Flow-Cells were treated for 6 h with 200 μL of U-MSNPs (25, 50, and 200 μg/mL) and urease (100 and 200 μg/mL), in both cases adding 100 mM urea. After the treatment, the biofilm was dyed with Live/Dead cells and observed under the confocal laser scanning microscope for biomass quantification with FIJI and COMSTAT2 software.

## Supporting Information Available

Characterization of enzyme-based mesoporous silica nanoparticles (Figure S1); enzyme activity evaluation of U-MSNPs and M-MSNPs over time (Figure S2); evaluating the bactericidal efficacy of the urease enzymatic products NH_4_^+^ and HCO_3_^–^ (Figure S3); evaluation of lysozyme activity (Figure S4); percentage of dead bacteria obtained from a live/dead assay (Figure S5); images corresponding to the live/dead assay (Figure S6); *E. coli* counts after 2 and 4 h of treatment with urease, U-MSNPs, L-MSNPs, and M-MSNPs (Figure S7); photograph of Petri plates at 10^3^ CFU dilution used to measure the effects of urease, U-MSNPs, L-MSNPs, and M-MSNPs against *E. coli* after 2 h (Figure S8); photograph of Petri plates at 10^3^ CFU dilution used to measure the effect of urease, U-MSNPs, L-MSNPs, and M-MSNPs against *E. coli* after 4 h (Figure S9) (PDF)
U-MSNP nanomotors in LB at 0 mM and 100 mM urea concentrations (Video S1) (AVI)
U-MSNP nanomotors in PBS at 0mM and 100 mM urea concentrations (Video S2) (AVI)
U-MSNP nanomotors in simulated urine at 0mM and100 mM urea concentrations (Video S3) (AVI)

## Supplementary Material

am1c00986_si_001.pdf

am1c00986_si_002.avi

am1c00986_si_003.avi

am1c00986_si_004.avi

## References

[ref1] Díez-Martínez R.; García-Fernández E.; Manzano M.; Martínez Á.; Domenech M.; Vallet-Regí M.; García P. Auranofin-Loaded Nanoparticles as a New Therapeutic Tool to Fight Streptococcal Infections. Sci. Rep. 2016, 6, 19525 10.1038/srep19525. 26776881 PMC4726118

[ref2] Taubes G. The Bacteria Fight Back. Science 2008, 321, 356–361. 10.1126/science.321.5887.356. 18635788

[ref3] Aslam B.; Wang W.; Arshad M. I.; Khurshid M.; Muzammil S.; Rasool M. H.; Nisar M. A.; Alvi R. F.; Aslam M. A.; Qamar M. U.; Salamat M. K. F.; Baloch Z. Antibiotic Resistance: A Rundown of a Global Crisis. In Infection and Drug Resistance; Dove Medical Press Ltd., 2018; pp 1645–1658. 10.2147/IDR.S173867 PMC6188119 30349322

[ref4] Kumar A.; Chen F.; Mozhi A.; Zhang X.; Zhao Y.; Xue X.; Hao Y.; Zhang X.; Wang P. C.; Liang X. J. Innovative Pharmaceutical Development Based on Unique Properties of Nanoscale Delivery Formulation. Nanoscale 2013, 5, 8307–8325. 10.1039/c3nr01525d. 23860639 PMC3934102

[ref5] Ventola C. L. The Nanomedicine Revolution: Part 1: Emerging Concepts. Pharm. Ther. 2012, 37, 512. PMC3462600 23066345

[ref6] Senapati S.; Mahanta A. K.; Kumar S.; Maiti P. Controlled Drug Delivery Vehicles for Cancer Treatment and Their Performance. Signal Transduction Targeted Ther. 2018, 3, 1–19. 10.1038/s41392-017-0004-3. PMC5854578 29560283

[ref7] Marchesan S.; Prato M. Nanomaterials for (Nano)Medicine. ACS Med. Chem. Lett. 2013, 4, 147–149. 10.1021/ml3003742. 24900637 PMC4027518

[ref8] Riehemann K.; Schneider S. W.; Luger T. A.; Godin B.; Ferrari M.; Fuchs H. Nanomedicine - Challenge and Perspectives. Angew. Chem., Int. Ed. 2009, 48, 872–897. 10.1002/anie.200802585. PMC4175737 19142939

[ref9] Barua S.; Mitragotri S. Challenges Associated with Penetration of Nanoparticles across Cell and Tissue Barriers: A Review of Current Status and Future Prospects. Nano Today 2014, 9, 223–243. 10.1016/j.nantod.2014.04.008. 25132862 PMC4129396

[ref10] Tu Y.; Peng F.; André A. A. M.; Men Y.; Srinivas M.; Wilson D. A. Biodegradable Hybrid Stomatocyte Nanomotors for Drug Delivery. ACS Nano 2017, 11, 1957–1963. 10.1021/acsnano.6b08079. 28187254 PMC5348104

[ref11] Llopis-Lorente A.; Garciá-Fernández A.; Murillo-Cremaes N.; Hortelaõ A. C.; Patinõ T.; Villalonga R.; Sancenón F.; Martínez-Máñez R.; Sánchez S. Enzyme-Powered Gated Mesoporous Silica Nanomotors for on-Command Intracellular Payload Delivery. ACS Nano 2019, 13, 12171–12183. 10.1021/acsnano.9b06706. 31580642

[ref12] Medina-Sánchez M.; Xu H.; Schmidt O. G. Micro- and Nano-Motors: The New Generation of Drug Carriers. Ther. Delivery 2018, 9, 303–316. 10.4155/tde-2017-0113. 29540126

[ref13] Chandrawati R.; Hosta-Rigau L.; Vanderstraaten D.; Lokuliyana S. A.; Städler B.; Albericio F.; Caruso F. Engineering Advanced Capsosomes: Maximizing the Number of Subcompartments, Cargo Retention, and Temperature-Triggered Reaction. ACS Nano 2010, 4, 1351–1361. 10.1021/nn901843j. 20192233

[ref14] Pijpers I. A. B.; Cao S.; Llopis-Lorente A.; Zhu J.; Song S.; Joosten R. R. M.; Meng F.; Friedrich H.; Williams D. S.; Sánchez S.; van Hest J. C. M.; Abdelmohsen L. K. E. A. Hybrid Biodegradable Nanomotors through Compartmentalized Synthesis. Nano Lett. 2020, 20, 4472–4480. 10.1021/acs.nanolett.0c01268. 32427492 PMC7291354

[ref15] Esteban-Fernández de Ávila B.; Angsantikul P.; Ramírez-Herrera D. E.; Soto F.; Teymourian H.; Dehaini D.; Chen Y.; Zhang L.; Wang J. Hybrid Biomembrane-Functionalized Nanorobots for Concurrent Removal of Pathogenic Bacteria and Toxins. Sci. Rob. 2018, 3, eaat0485 10.1126/scirobotics.aat0485. 33141704

[ref16] Esteban-Fernández de Ávila B.; Lopez-Ramirez M. A.; Mundaca-Uribe R.; Wei X.; Ramírez-Herrera D. E.; Karshalev E.; Nguyen B.; Fang R. H.; Zhang L.; Wang J. Multicompartment Tubular Micromotors Toward Enhanced Localized Active Delivery. Adv. Mater. 2020, 32, 2000091 10.1002/adma.202000091. 32419239

[ref17] Tang S.; Zhang F.; Gong H.; Wei F.; Zhuang J.; Karshalev E.; Esteban-Fernández de Ávila B.; Huang C.; Zhou Z.; Li Z.; Yin L.; Dong H.; Fang R. H.; Zhang X.; Zhang L.; Wang J. Enzyme-Powered Janus Platelet Cell Robots for Active and Targeted Drug Delivery. Sci. Rob. 2020, 5, eaba6137 10.1126/scirobotics.aba6137. 33022613

[ref18] Alapan Y.; Bozuyuk U.; Erkoc P.; Karacakol A. C.; Sitti M. Multifunctional Surface Microrollers for Targeted Cargo Delivery in Physiological Blood Flow. Sci. Rob. 2020, 5, eaba5726 10.1126/scirobotics.aba5726. 33022624

[ref19] Ma X.; Hortelão A. C.; Patiño T.; Sánchez S. Enzyme Catalysis To Power Micro/Nanomachines. ACS Nano 2016, 10, 9111–9122. 10.1021/acsnano.6b04108. 27666121 PMC5228067

[ref20] Dey K. K.; Zhao X.; Tansi B. M.; Méndez-Ortiz W. J.; Córdova-Figueroa U. M.; Golestanian R.; Sen A. Micromotors Powered by Enzyme Catalysis. Nano Lett. 2015, 15, 8311–8315. 10.1021/acs.nanolett.5b03935. 26587897

[ref21] Wilson D. A.; Nolte R. J. M.; van Hest J. C. M. Autonomous Movement of Platinum-Loaded Stomatocytes. Nat. Chem. 2012, 4, 268–274. 10.1038/nchem.1281. 22437710

[ref22] Abdelmohsen L. K. E. A.; Nijemeisland M.; Pawar G. M.; Janssen G.-J. A.; Nolte R. J. M.; van Hest J. C. M.; Wilson D. A. Dynamic Loading and Unloading of Proteins in Polymeric Stomatocytes: Formation of an Enzyme-Loaded Supramolecular Nanomotor. ACS Nano 2016, 10, 2652–2660. 10.1021/acsnano.5b07689. 26811982

[ref23] Wang L.; Hortelao A. C.; Huang X.; Sanchez S. Lipase-Powered Mesoporous Silica Nanomotors for Triglyceride Degradation. Angew. Chem., Int. Ed. 2019, 58, 7992–7996. 10.1002/anie.201900697. 30990243

[ref24] Wang L.; Marciello M.; Estévez-Gay M.; Soto Rodriguez P. E. D.; Luengo Morato Y.; Iglesias-Fernández J.; Huang X.; Osuna S.; Filice M.; Sánchez S. Enzyme Conformation Influences the Performance of Lipase-powered Nanomotors. Angew. Chem., Int. Ed. 2020, 59, 21080–21087. 10.1002/anie.202008339. 32755070

[ref25] Ma X.; Jannasch A.; Albrecht U.-R.; Hahn K.; Miguel-López A.; Schäffer E.; Sánchez S. Enzyme-Powered Hollow Mesoporous Janus Nanomotors. Nano Lett. 2015, 15, 7043–7050. 10.1021/acs.nanolett.5b03100. 26437378

[ref26] Ma X.; Hortelao A. C.; Miguel-López A.; Sánchez S. Bubble-Free Propulsion of Ultrasmall Tubular Nanojets Powered by Biocatalytic Reactions. J. Am. Chem. Soc. 2016, 138, 13782–13785. 10.1021/jacs.6b06857. 27718566 PMC5228068

[ref27] Patiño T.; Arqué X.; Mestre R.; Palacios L.; Sánchez S. Fundamental Aspects of Enzyme-Powered Micro- and Nanoswimmers. Acc. Chem. Res. 2018, 51, 2662–2671. 10.1021/acs.accounts.8b00288. 30346732

[ref28] Díez P.; Esteban-Fernández de Ávila B.; Ramírez-Herrera D. E.; Villalonga R.; Wang J. Biomedical Nanomotors: Efficient Glucose-Mediated Insulin Release. Nanoscale 2017, 9, 14307–14311. 10.1039/C7NR05535H. 28930338

[ref29] Hortelão A. C.; Patiño T.; Perez-Jiménez A.; Blanco À.; Sánchez S. Enzyme-Powered Nanobots Enhance Anticancer Drug Delivery. Adv. Funct. Mater. 2018, 28, 1705086 10.1002/adfm.201705086.

[ref30] Gao W.; Esteban-Fernández de Ávila B.; Zhang L.; Wang J. Targeting and Isolation of Cancer Cells Using Micro/Nanomotors. Adv. Drug Delivery Rev. 2018, 125, 94–101. 10.1016/j.addr.2017.09.002. PMC5844782 28893551

[ref31] Esteban-Fernández de Ávila B.; Ramírez-Herrera D. E.; Campuzano S.; Angsantikul P.; Zhang L.; Wang J. Nanomotor-Enabled PH-Responsive Intracellular Delivery of Caspase-3: Toward Rapid Cell Apoptosis. ACS Nano 2017, 11, 5367–5374. 10.1021/acsnano.7b01926. 28467853 PMC5894870

[ref32] Esteban-Fernández de Ávila B.; Angsantikul P.; Li J.; Angel Lopez-Ramirez M.; Ramírez-Herrera D. E.; Thamphiwatana S.; Chen C.; Delezuk J.; Samakapiruk R.; Ramez V.; Zhang L.; Wang J. Micromotor-Enabled Active Drug Delivery for in Vivo Treatment of Stomach Infection. Nat. Commun. 2017, 8, 272 10.1038/s41467-017-00309-w. 28814725 PMC5559609

[ref33] Stanton M. M.; Park B.-W.; Vilela D.; Bente K.; Faivre D.; Sitti M.; Sanchez S. Magnetotactic Bacteria Powered Biohybrids Target *E. coli* Biofilms. ACS Nano 2017, 11, 9968–9978. 10.1021/acsnano.7b04128. 28933815

[ref34] Kiristi M.; Singh V. V.; Esteban-Fernández de Ávila B.; Uygun M.; Soto F.; Aktaş Uygun D.; Wang J. Lysozyme-Based Antibacterial Nanomotors. ACS Nano 2015, 9, 9252–9259. 10.1021/acsnano.5b04142. 26308491

[ref35] Bhuyan T.; Simon A. T.; Maity S.; Kumar Singh A.; Sankar Ghosh S.; Bandyopadhyay D. Magnetotactic T-Budbots to Kill-n-Clean Biofilms. ACS Appl. Mater. Interfaces 2020, 12, 43352–43364. 10.1021/acsami.0c08444. 32864951

[ref36] Villa K.; Viktorova J.; Plutnar J.; Ruml T.; Hoang L.; Pumera M. Chemical Microrobots as Self-Propelled Microbrushes against Dental Biofilm. Cell Rep. Phys. Sci. 2020, 1, 100181 10.1016/j.xcrp.2020.100181.

[ref37] Hoop M.; Shen Y.; Chen X.-Z.; Mushtaq F.; Iuliano L. M.; Sakar M. S.; Petruska A.; Loessner M. J.; Nelson B. J.; Pané S. Magnetically Driven Silver-Coated Nanocoils for Efficient Bacterial Contact Killing. Adv. Funct. Mater. 2016, 26, 1063–1069. 10.1002/adfm.201504463.

[ref38] Wu Y.; Song Z.; Deng G.; Jiang K.; Wang H.; Zhang X.; Han H. Gastric Acid Powered Nanomotors Release Antibiotics for In Vivo Treatment of *Helicobacter pylori* Infection. Small 2021, 17, 2006877 10.1002/smll.202006877. 33619851

[ref39] Delezuk J. A. M.; Ramírez-Herrera D. E.; Esteban-Fernández de Ávila B.; Wang J. Chitosan-based water-propelled micromotors with strong antibacterial activity. Nanoscale 2017, 9, 2195–2200. 10.1039/C6NR09799E. 28134392

[ref40] Hortelão A. C.; Carrascosa R.; Murillo-Cremaes N.; Patiño T.; Sánchez S. Targeting 3D Bladder Cancer Spheroids with Urease-Powered Nanomotors. ACS Nano 2019, 13, 429–439. 10.1021/acsnano.8b06610. 30588798

[ref41] Malamud D.; Drysdale J. W. Isoelectric points of proteins: A table. Anal. Biochem. 1978, 86, 620–647. 10.1016/0003-2697(78)90790-X. 26290

[ref42] Abeyrathne E. D. N. S.; Lee H. Y.; Ahn D. U. Sequential separation of lysozyme, ovomucin, ovotransferrin,and ovalbumin from egg white. Poult. Sci. 2014, 93, 1001–1009. 10.3382/ps.2013-03403. 24706978

[ref43] Ragland S. A.; Criss A. K. From bacterial killing to immune modulation: Recent insights into the functions of lysozyme. PLoS Pathog. 2017, 13, e1006512 10.1371/journal.ppat.1006512. 28934357 PMC5608400

[ref44] Diez-Gonzalez F.; Jarvis G. N.; Adamovich D. A.; Russell J. B. Use of Carbonate and Alkali To Eliminate *Escherichia coli* from Dairy Cattle Manure. Environ. Sci. Technol. 2000, 34, 1275–1279. 10.1021/es9910356.

[ref45] Jarvis G. N.; Fields M. W.; Adamovich D. A.; Arthurs C. E.; Russell J. B. The mechanism of carbonate killing of *Escherichia coli*. Lett. Appl. Microbiol. 2001, 33, 196–200. 10.1046/j.1472-765x.2001.00976.x. 11555203

[ref46] Russell J. B.; Jarvis G. N. Practical Mechanisms for Interrupting the Oral-fecal Lifecycle of *Escherichia coli*. J. Mol. Microbiol. Biotechnol. 2001, 3, 265–272. 11321582

[ref47] Park G. W.; Diez-Gonzalez F. Utilization of carbonate and ammonia-based treatments to eliminate *Escherichia coli* O157:H7 and Salmonella Typhimurium DT104 from cattle manure. J. Appl. Microbiol. 2003, 94, 675–685. 10.1046/j.1365-2672.2003.01899.x. 12631203

[ref48] Vilcacundo R.; Méndez P.; Reyes W.; Romero H.; Pinto A.; Carrillo W. Antibacterial activity of hen egg white lysozyme denatured by thermal and chemical treatments. Sci. Pharm. 2018, 86, 48 10.3390/scipharm86040048. 30380756

[ref49] Wild P.; Gabrieli A.; Schraner E. M.; Pellegrini A.; Thomas U.; Frederik P. M.; Stuart M. C. A.; Von Fellenberg R. Reevaluation of the effect of lysoyzme on *Escherichia coli* employing ultrarapid freezing followed by cryoelectronmicroscopy or freeze substitution. Microsc. Res. Tech. 1997, 39, 297–304. 10.1002/(SICI)1097-0029(19971101)39:3<297::AID-JEMT8>3.0.CO;2-H. 9372501

[ref50] Purcell E. M. The shape of low Reynolds number jets, Cavity Low Reynolds Number. Phys. Fluids 1977, 45, 1631.

[ref51] Ma X.; Wang X.; Hahn K.; Sánchez S. Motion Control of Urea-Powered Biocompatible Hollow Microcapsules. ACS Nano 2016, 10, 3597–605. 10.1021/acsnano.5b08067. 26863183

[ref52] Patiño T.; Feiner-Gracia N.; Arqué X.; Miguel-López A.; Jannasch A.; Stumpp T.; Schäffer E.; Albertazzi L.; Sánchez S. Influence of Enzyme Quantity and Distribution on the Self-Propulsion of Non-Janus Urease-Powered Micromotors. J. Am. Chem. Soc. 2018, 140, 7896–7903. 10.1021/jacs.8b03460. 29786426

[ref53] Anfora A. T.; Halladin D. K.; Haugen B. J.; Welch R. A. Uropathogenic *Escherichia coli* CFT073 is adapted to acetatogenic growth but does not require acetate during murine urinary tract infection. Infect. Immun. 2008, 76, 5760–5767. 10.1128/IAI.00618-08. 18838520 PMC2583553

[ref54] Blanco-Cabra N.; Vega-Granados K.; Moya-Andérico L.; Vukomanovic M.; Parra A.; Álvarez De Cienfuegos L.; Torrents E. Novel Oleanolic and Maslinic Acid Derivatives as a Promising Treatment against Bacterial Biofilm in Nosocomial Infections: An in Vitro and in Vivo Study. ACS Infect. Dis. 2019, 5, 1581–1589. 10.1021/acsinfecdis.9b00125. 31268675

[ref55] Dunderdale G.; Ebbens S.; Fairclough P.; Howse J. Importance of Particle Tracking and Calculating the Mean-Squared Displacement in Distinguishing Nanopropulsion from Other Processes. Langmuir 2012, 28, 10997–11006. 10.1021/la301370y. 22731393

