# Supplementary material for: Drug-Free Enzyme-Based Bactericidal Nanomotors against Pathogenic Bacteria
Source: ACS Appl Mater Interfaces. 2021 Mar 26;13(13):14964–73. doi: 10.1021/acsami.1c00986 (PMC8478280; doi:10.1021/acsami.1c00986)
Supplement: Supplementary file 1 — am1c00986_si_001.pdf [file am1c00986_si_001.pdf]

Drug-free enzyme-based bactericidal nanomotors against pathogenic bacteria

Diana Vilela,[‡†] Nuria Blanco-Cabra,[§] Ander Eguskiza,[‡] Ana C. Hortelao,[‡] Eduard Torrents[§£] and Samuel Sanchez[‡¥*]*

‡ Smart nano-bio-devices. Institute for Bioengineering of Catalonia (IBEC), The Barcelona Institute of Science and Technology (BIST). Baldiri Reixac 10-12, 08028 Barcelona Spain.

§ Bacterial infections: antimicrobial therapies. Institute for Bioengineering of Catalonia (IBEC), The Barcelona Institute of Science and Technology (BIST) Baldiri Reixac 10-12, 08028 Barcelona Spain.

£ Microbiology Section, Department of Genetics, Microbiology and Statistics Faculty of Biology. University of Barcelona 643 Diagonal Ave., 08028, Barcelona, Spain.

¥ Institució Catalana de Recerca i Estudis Avancats (ICREA). Pg. Lluís Companys 23, 08010, Barcelona, Spain

* E-mail: divilela@ucm.es and ssanchez@ibecbarcelona.eu

KEYWORDS

Enzymatic nanomotors • biofilms • *E. coli* • infections • nanomachines • self-propulsion

TABLE OF CONTENTS

Figure S1. Characterization of enzyme-based mesoporous silica nanoparticles. (A) Calibration plot and amount of protein bonded to each type of MSNP (1mg/mL) for absorbance of 0.319 + 5.43×10^{-4}[Protein] and a protein concentration range of 2.5-500 μg/mL the slight blue tinge in some samples were obtained once Coomassie brilliant blue G had reacted with the proteins in each sample. (B) Enzymatic activity of urease bound to three different types of MSNP (N=3, error bars indicate SE).

Figure S2. Enzyme activity of U-MSNPs and M-MSNPs during 14 days of storage at 4ºC in PBS with pH 7.4.

Figure S3. Evaluating the bactericidal efficacy of different concentrations (0-50 mM) of the urease enzymatic products NH_4^+ and HCO_3^- after 1 hour of

incubation with *E. coli* strain MG1655. (A) Fluorescence images of dead (red) and live (green) bacteria after 1h of incubation of 1x10^8 CFU/mL of *E. coli* with NH_4^+ and HCO_3^- (scale bars = 50 μm). (B) Percentage of dead bacteria as calculated from the fluorescence images in A (N=3, error bars represent SE).

Figure S4. Evaluation of lysozyme activity: live bacteria obtained from live-dead assay after 2 hours of incubation of (A) lysozyme with *M. lysodeikticus* (0.1 mg/mL), (B) lysozyme with *E. coli,* and (C) L-MSNPs with *E. coli*.

Figure S5. Percentage of dead bacteria obtained from a live-dead assay after 2 hours of 1x10^8 CFU/mL *E. coli* treated with 12.5 μg/mL (minimum inhibitory concentration, MIC_{50}).

Figure S6. Images corresponding to the live/dead assay for the protein-modified MSNPs and controls after 2 hours of treatment.

Figure S7. *E. coli* counts (log10 CFU/mL) after 2 and 4 hours of treatment with 12.5 μg/mL (MIC_{50}) urease, U-MSNPs, L-MSNPs, and M-MSNPs, including the controls.

Figure S8. Photograph of petri plates at 10^3 CFU dilution used to measure the effects of urease, U-MSNPs, L-MSNPs, and M-MSNPs against *E. coli* after 2 hours.

Figure S9. Photograph of petri plates at 10^3 CFU dilution used to measure the effect of urease, U-MSNPs, L-MSNPs, and M-MSNPs against *E. coli* after 4 hours.

Video S1. U-MSNP nanomotors in LB at 0 mM and 100 mM urea concentrations.

Video S2. U-MSNP nanomotors in PBS at 0mM and 100 mM urea concentrations.

Video S3. U-MSNP nanomotors in simulated urine at 0mM and100 mM urea concentrations.

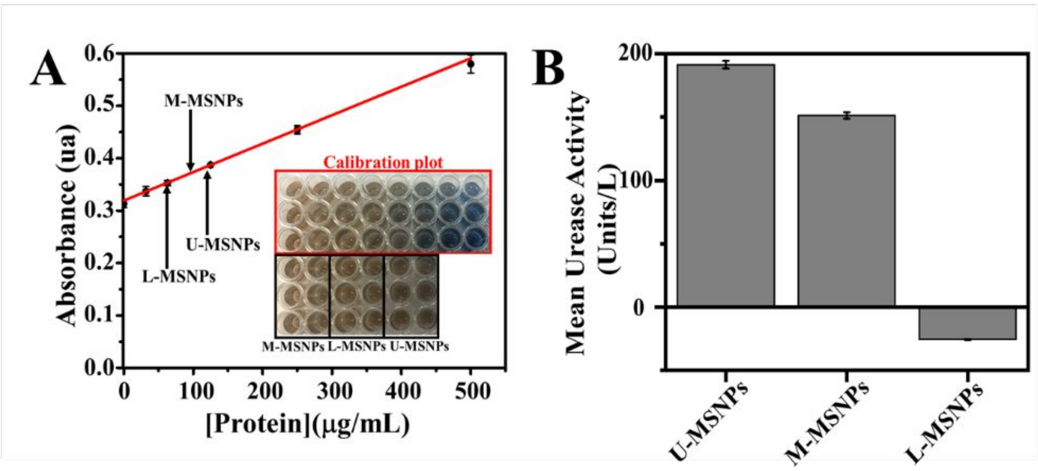

Figure S1. Characterization of enzyme-based mesoporous silica nanoparticles. (A) Calibration plot and amount of protein bonded to each type of MSNP (1mg/mL) for absorbance of 0.319 + 5.43×10^{-4}[Protein] and a protein concentration range of 2.5-500 μg/mL the slight blue tinge in some samples were obtained once Coomassie brilliant blue G had reacted with the proteins in each sample. (B) Enzymatic activity of urease bound to three different types of MSNP (N=3, error bars indicate SE).

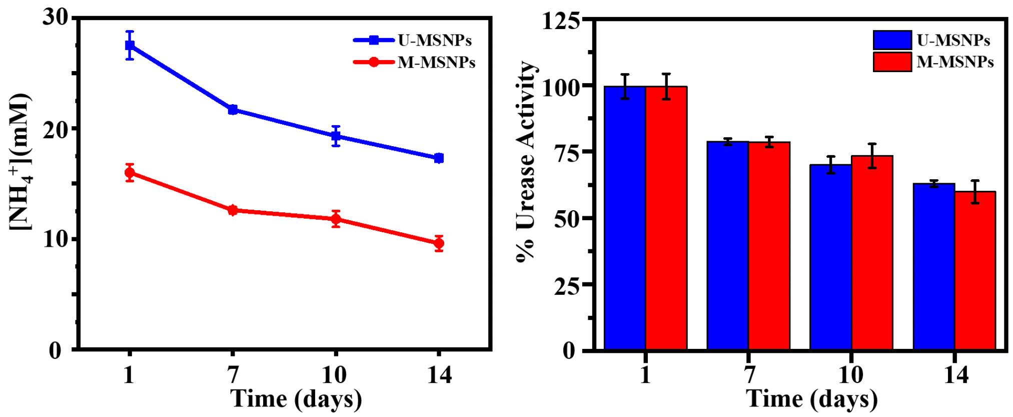

Figure S2. Enzyme activity of U-MSNPs and M-MSNPs during 14 days of storage at 4ºC in PBS with pH 7.4.

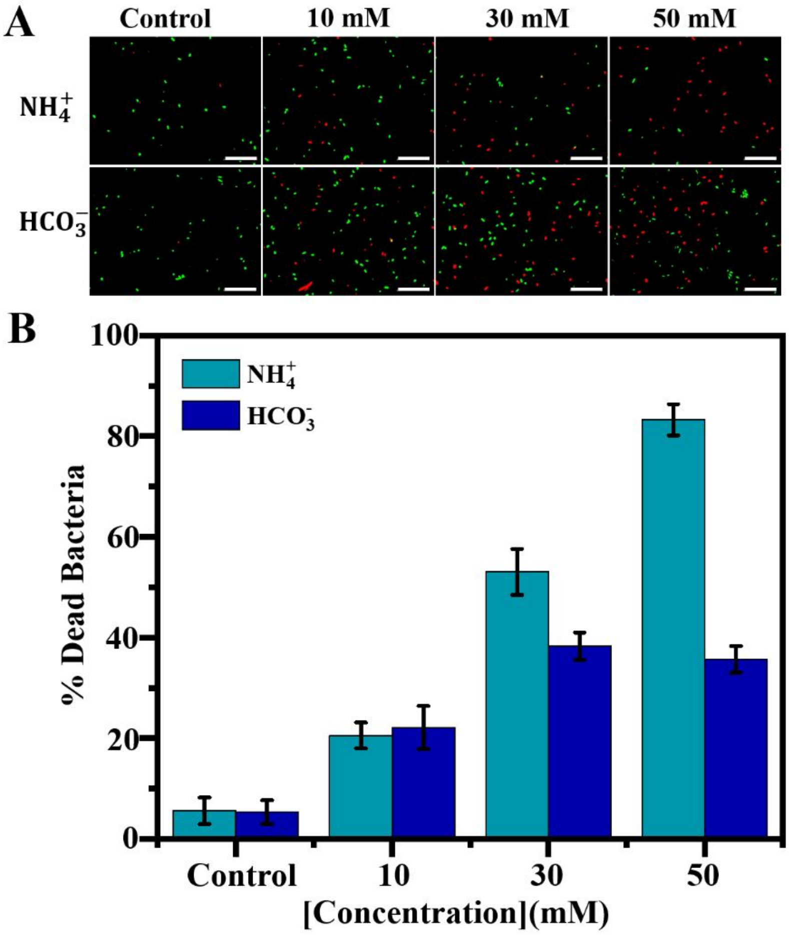

Figure S3. Evaluating the bactericidal efficacy of different concentrations (0-50 mM) of the urease enzymatic products NH_4^+ and HCO_3^- after 1 hour of incubation with *E. coli* strain MG1655. (A) Fluorescence images of dead (red) and live (green) bacteria after 1h of incubation of 1x10^8 CFU/mL of *E. coli* with NH_4^+ and HCO_3^- (scale bars = 50 μm). (B) Percentage of dead bacteria as calculated from the fluorescence images in A (N=3, error bars represent SE).

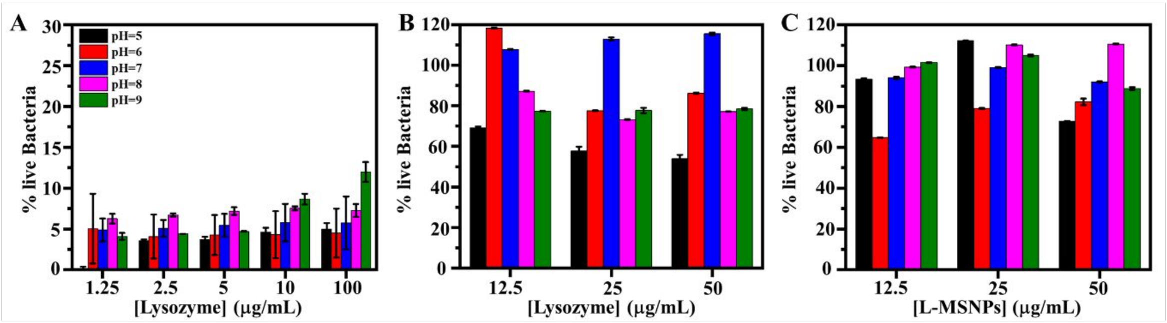

Figure S4. Evaluation of lysozyme activity: live bacteria obtained from live-dead assay after 2 hours of incubation of (A) lysozyme with *M. lysodeikticus* (0.1 mg/mL), (B) lysozyme with *E. coli,* and (C) L-MSNPs with *E. coli.*

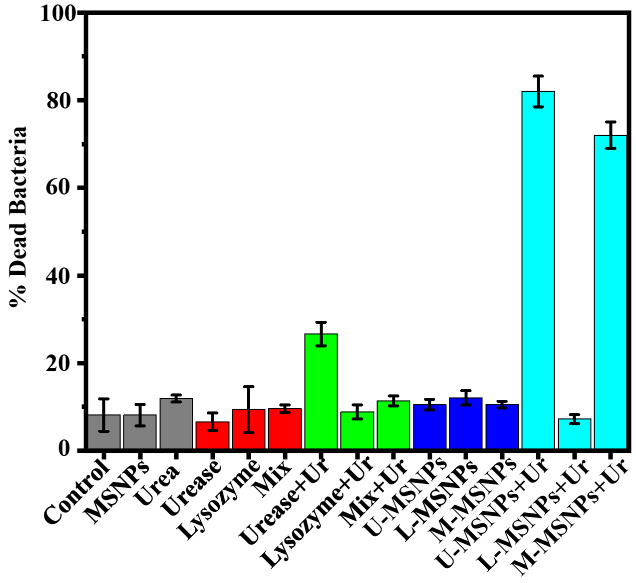

Figure S5. Percentage of dead bacteria obtained from a live-dead assay after 2 hours of 1x10^8 CFU/mL *E. coli* treated with 12.5 μg/mL (minimum inhibitory concentration, MIC_{50}).

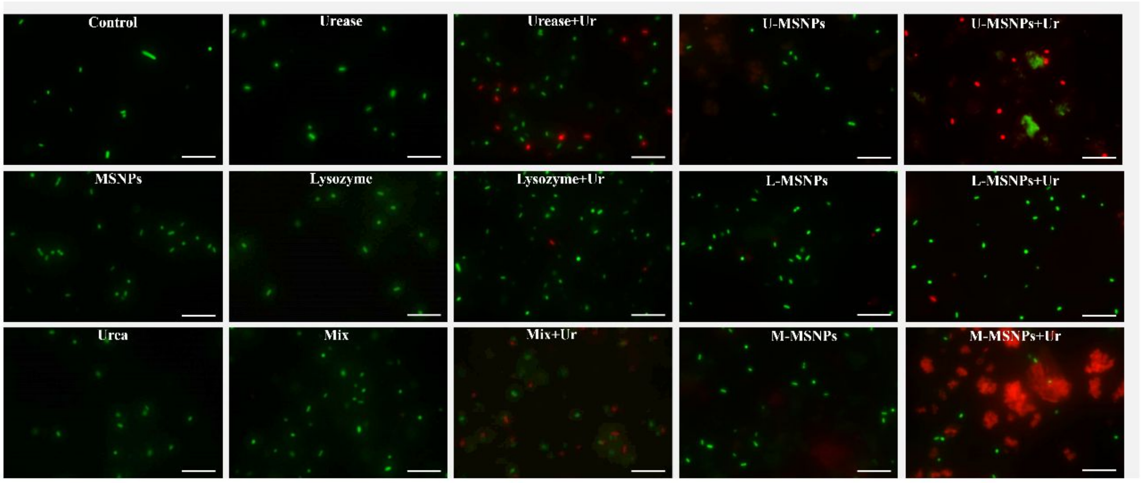

Figure S6. Images corresponding to the live/dead assay for the protein-modified MSNPs and controls after 2 hours of treatment.

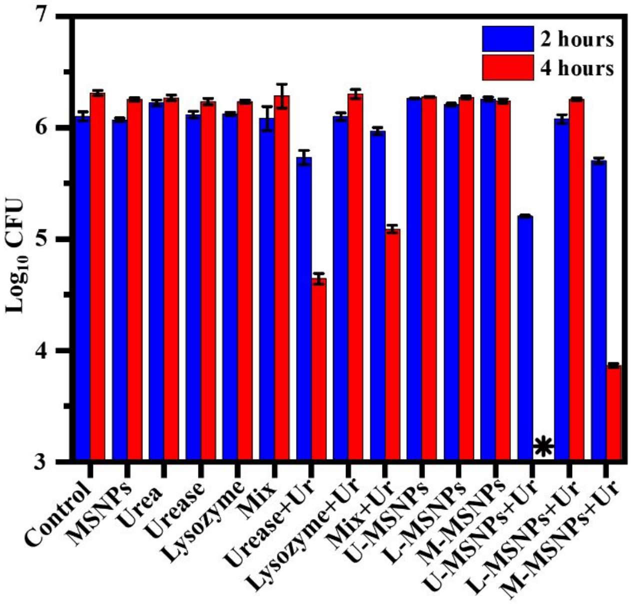

Figure S7. *E. coli* counts (log10 CFU/mL) after 2 and 4 hours of treatment with 12.5 μg/mL (MIC_{50}) urease, U-MSNPs, L-MSNPs, and M-MSNPs, including the controls.

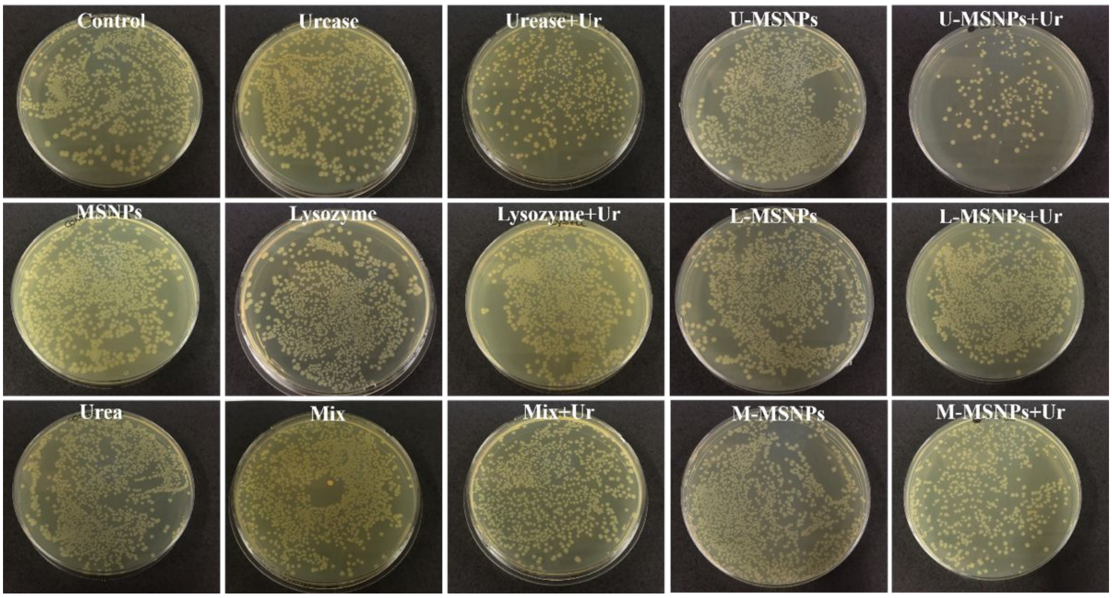

Figure S8. Photograph of petri plates at 10^3 CFU dilution used to measure the effects of urease, U-MSNPs, L-MSNPs, and M-MSNPs against *E. coli* after 2 hours.

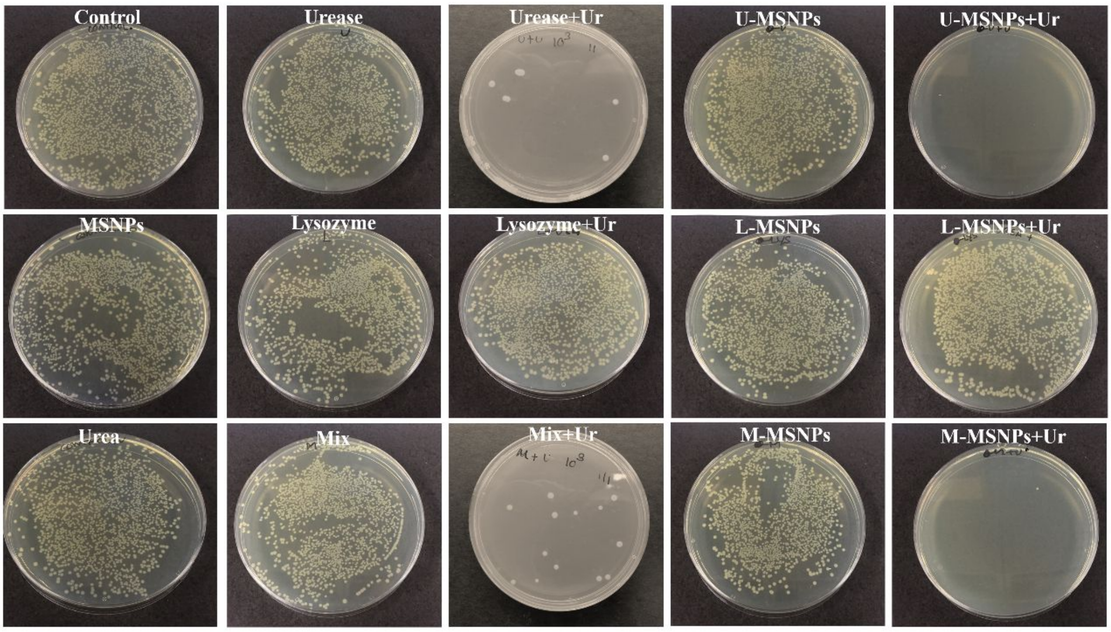

Figure S9. Photograph of petri plates at 10^3 CFU dilution used to measure the effect of urease, U-MSNPs, L-MSNPs, and M-MSNPs against *E. coli* after 4 hours.

AUTHOR INFORMATION

Corresponding Author

*E-mail: ssanchez@ibecbarcelona.eu, divilela@ucm.es

ACKNOWLEDGEMENTS

The research leading to the results presented here has received funding from the European Research Council (ERC) under the European Union's Horizon 2020 research and innovation programme (grant agreement No 866348)" and from the Spanish MINECO (CTQ2015-68879-R (MICRODIA) and CTQ2015-72471-EXP (Enzwim)). SS acknowledges financial support from the BBVA Foundation through the MEDIROBOTS project as well as the CERCA program by the Generalitat de Catalunya. D V acknowledges financial support by the European Commission under a Horizon 2020 Marie Skłodowska-Curie Action COFUND scheme (grant agreement no. 712754) and by the Severo Ochoa programme of the Spanish Ministry of Economy and Competitiveness (grant no. SEV-2014-0425). ACH wishes to thank MINECO for the Severo Ochoa fellowship. ET acknowledges support from La Caixa Foundation, Ministerio de Ciencia, Innovación y Universidades (MCIU), Agencia Estatal de Investigación (AEI), and Fondo Europeo de Desarrollo Regional (FEDER) (RT12018-098573-B-100), and

from the CERCA programme/Generalitat de Catalunya (2017 SGR01079). The authors also thank the CERCA Programme/Generalitat de Catalunya for financial support and A. M. López for developing the Python code used for the motion analysis. Editorial assistance, in the form of language editing and correction, was provided by XpertScientific Editing and Consulting Services.
